# Cell-Free DNA, Neutrophil extracellular traps (NETs), and Endothelial Injury in Coronavirus Disease 2019– (COVID-19–) Associated Acute Kidney Injury

**DOI:** 10.1155/2022/9339411

**Published:** 2022-04-22

**Authors:** Brandon Michael Henry, Maria Helena Santos de Oliveira, Isaac Cheruiyot, Justin Benoit, James Rose, Emmanuel J. Favaloro, Giuseppe Lippi, Stefanie Benoit, Naomi Pode Shakked

**Affiliations:** ^1^Division of Nephrology and Hypertension, Cincinnati Children's Hospital Medical Center, Cincinnati, OH, USA; ^2^Department of Statistics, Federal University of Parana, Curitiba, Brazil; ^3^School of Medicine, University of Nairobi, Kenya; ^4^Department of Emergency Medicine, University of Cincinnati, Cincinnati, OH, USA; ^5^Haematology, Sydney Centres for Thrombosis and Haemostasis, Institute of Clinical Pathology and Medical Research (ICPMR), NSW Health Pathology, Westmead Hospital, Westmead, NSW, Australia; ^6^Section of Clinical Biochemistry, Department of Neurosciences, Biomedicine and Movement Sciences, University of Verona, Italy; ^7^Division of Bone Marrow Transplantation and Immunodeficiency, Cincinnati Children's Hospital Medical Center, Cincinnati, OH, USA; ^8^Department of Pediatrics, University of Cincinnati College of Medicine, Cincinnati, Ohio, USA; ^9^The Sheba Talpiot Medical Leadership Program, Sheba Medical Center, Israel; ^10^Sackler School of Medicine, Tel Aviv University, Tel Aviv, Israel

## Abstract

*Introduction*: Neutrophil extracellular traps (NETs) release (i.e., NETosis) has been recently implicated in the pathomechanism underlying severe end-organ damage in Coronavirus Disease 2019 (COVID-19) and could present a novel therapeutic target. We aimed to determine whether circulating levels of cell-free DNA (cfDNA), a surrogate for NETosis, may be associated with the development of acute kidney injury (AKI), a major contributor to poor outcomes and mortality in COVID-19. *Methods*: Blood samples were collected prospectively from adult patients infected with SARS-CoV-2 presenting to the emergency department (ED). Circulating levels of cfDNA were quantified from patients' serum. Further assessment of correlations between cfDNA levels and markers of AKI (i.e., serum creatinine (SCr), cystatin C, neutrophil gelatinase–associated lipocalin (NGAL)), biomarkers of thrombotic microangiopathy and of inflammation in patients' serum was performed. *Results*: Fifty-one COVID-19 patients were enrolled. cfDNA levels were found to be significantly higher in those who developed severe AKI (*p* < 0.001) and those needing renal replacement therapy (*p* = 0.020). cfDNA positively correlated with ED SCr, NGAL, cystatin C, neutrophil count, neutrophil-to-lymphocyte ratio, C3a, C5a, Scb5-9, IL-6, IL-8, IL-10, TNF-*α*, LDH, CRP, ferritin, and fibrinogen and negatively correlated with ADAMTS13/von-Willebrand factor ratio and lymphocyte count. In a multivariate logistic regression, a one-unit increase in cfDNA value was associated with 4.6% increased odds of severe AKI (OR = 1.046; *p* = 0.040). Finally, cfDNA significantly correlated with established NETs components, myeloperoxidase, and neutrophil elastase. *Conclusion*: Intravascular NETosis could be an important contributing factor in the development of microthrombosis and COVID-19-associated AKI. Further research is urgently needed to understand the role of NETosis in COVID-19 and evaluate therapeutic avenues for targeting this process.

## 1. Introduction

Recent autopsy studies have described the presence of diffuse endotheliitis and extensive microthrombi in pulmonary, cardiac, and renal microvasculature of patients with the coronavirus disease 2019 (COVID-19) [1–4]. These observations, coupled with laboratory findings of low ADAMTS13 activity (i.e., a disintegrin and metalloproteinase with a thrombospondin type 1 motif, member 13, a circulating enzyme which cleaves von Willebrand factor (VWF) multimers reducing their activity) to von VWF antigen (VWF:Ag) ratio [5], elevated lactate dehydrogenase (LDH) [6] and interleukin-6 (IL-6), thrombocytopenia [7, 8], and anemia [9], suggest that COVID-19 may be associated with a thrombotic microangiopathy– (TMA–) like condition. This phenomenon is thought to be a major driver of morbidity and mortality in patients with severe COVID-19 and results in end-organ damage such as acute lung injury (ALI) [10], acute cardiac injury (ACI) [11], and acute kidney injury (AKI) [12, 13]. The mechanisms of the pathogenesis of TMA in COVID-19, however, have yet to be fully elucidated.

A substantial body of evidence suggests that neutrophils play a central role in the pathogenesis of the TMA-like phenomenon in COVID-19 patients, through dysregulated formation of neutrophil extracellular traps (NETs) [14, 15]. NETs are extracellular fibers composed of double-stranded deoxyribonucleic acid (dsDNA) with histones and enzymes such as elastase and myeloperoxidase (MPO) [16]. Initially described by Brinkman et al. [17] in 2004 as a bactericidal mechanism of neutrophils, NETs have been implicated in the formation of microthrombi by trapping platelets, thus facilitating their activation, adhesion, and aggregation [18]. They are also rich in tissue factor (TF), which can activate the coagulation cascade [19]. Furthermore, NETs have been shown to impair the fibrinolytic pathway in patients with non-COVID-19 septic shock, resulting in persistence of microthrombi within capillary networks [20], and suggested to play a role in sepsis-induced multiorgan failure [21] and COVID-19–induced sepsis [22]. While NETosis is growingly recognized as a substantial player in the pathophysiology of end-organ damage in COVID-19 [23], the predictive role of NETosis markers and COVID-19–related severe AKI has yet to be evaluated.

Herein, we carried out a prospective observational study to determine whether circulating levels of cell-free DNA (cfDNA), a surrogate of NETs release (NETosis) and tissue damage, may be associated with end-organ injury, specifically COVID-19–induced AKI. For this purpose, we have analyzed cfDNA serum levels and their correlation with markers of AKI serum creatinine (SCr), cystatin C, and neutrophil gelatinase associated lipocalin (NGAL), as well as with biomarkers of TMA and inflammation (ADAMTS13 activity/VWF:Ag (ADAMTS13:VWF) ratio, complement factor 3a (C3a), complement factor 5 a (C5a), soluble membrane attack complex (Scb5-9), LDH, IL-6, IL-8, IL-10, tumor necrosis factor-alpha (TNF- *α*), C-reactive protein (CRP), ferritin, fibrinogen, as wells as neutrophil, lymphocyte, and platelet counts). Finally, we further validated cfDNA as a surrogate of NETosis by analyzing its correlation with serum levels of NETs components myeloperoxidase (MPO) and neutrophil elastase.

## 2. Materials and Methods

### 2.1. Study Design

This was a prospective observational study. Adults (≥18 years old) presenting to the emergency department (ED) of the University of Cincinnati Medical Center with respiratory symptoms at triage suggestive of COVID-19 and with a positive reverse transcription polymerase chain reaction (RT-PCR) test for COVID-19 via nasopharyngeal swab were enrolled. This study was approved by the University of Cincinnati Institutional Review board (IRB) (IRB protocol ID, 2020-0278; approval date, 03/17/2020) and performed under a waiver of informed consent.

### 2.2. Sample Collection, Processing, and Biomarker Measurements

Blood samples in EDTA and/or heparin-containing tubes were collected via routine draws for clinical indications in the ED. Following collection, the samples were centrifuged at 2,000 g for 15 min at 4 °C and subsequently frozen at −80 °C until measurement.

Serum creatinine was assayed using a kinetic alkaline picrate (modified Jaffe) method on either a Beckman Coulter AU480 Chemistry Analyzer (Brea, CA, USA) or a Beckman Coulter AU5822 Chemistry Analyzer. Serum cystatin C (sCysC) was measured using a Siemens N Latex Cystatin C assay (REF: OQNM19) on BN II System. Serum NGAL (sNGAL) was assayed with The NGAL Test (BioPorto Diagnostics A/S, Hellerup, Denmark), a particle-enhanced turbidimetric immunoassay, also using a BN II System.

Complete blood counts with differential were quantified using a Beckman Coulter UniCel DxH 800 Cellular Analysis System (Brea, CA, USA). Plasma concentrations of fibrinogen, ferritin, and C-reactive protein (CRP) were measured by immunonephelometry on a Behring Nephelometer II (BNII, Siemens Medical Solutions USA, Inc., Malvern, PA, USA). Plasma concentrations of IL-6, IL-8, IL-10, and tumor necrosis factor-*α* (TNF-*α*) were measured using Meso Scale Discovery (MSD) U-Plex assay (Rockville, Maryland, USA). Quidel (San Diego, CA, USA) MicroVue ELISA kits were used to measure the complement components: C3a, C5a, and sC5b-9.

Lactate dehydrogenase was assessed with a Dimension RxL Max Integrated Chemistry System (Siemens Medical Solutions USA, Inc, Malvern, PA, USA). VWF:Ag was assayed using a Technozym ELISA (Diapharma, West Chester, OH, USA), while ADAMTS13 activity was measured using a fluorescence resonance energy transfer (FRET) assay (Immucor, Inc, Peachtree Corners, GA, USA). Neutrophil elastase and MPO levels were measured using Human PMN Elastase ELISA kit (Abcam, USA) and Human Myeloperoxidase Quantikine ELISA kit (R&D Systems, MN, USA), respectively, as previously described [24, 25]. All of the assays used in the investigation were performed according to the manufacturers' instructions and recommendations.

### 2.3. Quantification of cfDNA

Circulating levels of cfDNA was quantified from patients' serum using Quant-iT PicoGreen dsDNA Reagent and Kit (Thermo Fisher Scientific, Waltham, MA, USA) according to the manufacturer's instructions, with the exception that 15 *μ*L of patient serum was added to 185 *μ*L of the PicoGreen reagent solution for total reaction volume of 200 *μ*L in a 96-well plate. Samples were excited at 485 nm, and fluorescence intensity was measured at 520 nm on a Synergy H1 Reader (BioTek Instruments, Inc., Winooski, VT, USA).

### 2.4. Data Collection, Outcomes, and Study Definitions

Study data was prospectively recorded from patients' electronic medical records (EMR) and immediately recorded into a REDCap (Research Electronic Data Capture) database. Variables extracted included patient characteristics and demographics, past medical history, clinical course up to 30 days after initial contact in the ED, outcomes, and SARS-CoV-2–related complications. Data extraction was performed by a research physician, with selected records checked for accuracy by a second physician.

The primary outcome of interest was the development of severe AKI, defined as Kidney Disease Improving Global Outcomes (KDIGO) Stage 2 + 3 according to SCr criteria [26]. The secondary outcome was the need for renal replacement therapy (RRT).

### 2.5. Statistical Analysis

The analysis of the data was carried out using R (version 4.0.2, R Foundation for Statistical Computing, Vienna, Austria). Categorical data were reported as frequencies (%), while continuous data were reported as median and interquartile range (IQR). Comparison of cfDNA between patients with/without severe AKI and patients needing/not needing RRT was carried out using the Mann–Whitney *U* test. The diagnostic performance of cfDNA for predicting severe AKI and the need for RRT was assessed with receiver operating characteristics (ROC) curve analysis, by calculation of the area under the curve (AUC) and its 95% confidence interval (95% CI). Logistic regression was performed to estimate the effect of cfDNA concentration on primary and secondary outcomes, adjusting for age, sex, and comorbidities, and to calculate adjusted odds ratios (ORs) with the corresponding 95% Wald CI. Additionally, correlation between cfDNA and markers of AKI, TMA, and inflammation was performed using Spearman's rank correlation coefficient.

## 3. Results

### 3.1. Patient Characteristics

A total of 51 COVID-19 patients were included. The median age was 50.5 (IQR: 39.3-66.0) years, and 57.7% were males. Their baseline characteristics and comorbidities are shown in Table 1. At admission, 19 patients (37.3%) had mild COVID-19, 26 (51.0%) had moderate disease, and 6 (11.8%) had severe disease. At peak, 19 (37.3%) had mild disease, 16 (31.4%) had moderate disease, and 16 (31.4%) had severe disease. A total of 12 (23.5%) COVID-19 patients developed severe AKI, 8 of whom (66.6%) needed RRT.

### 3.2. Relationship between cfDNA Levels and Severe AKI/Need for RRT

**Figure 1:**
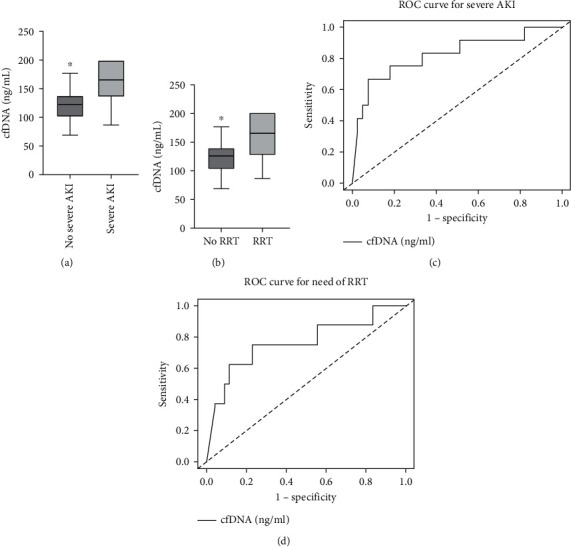
Cell-free DNA (cfDNA) levels in COVID-19 patients based on severity of acute kidney injury (AKI) (a) and need for renal replacement therapy (RRT) (b); receiver operating characteristic curve (ROC) of cfDNA for severe AKI (c, left) (area under the curve (AUC) = 0.82 (95% CI: 0.67-0.97)); and ROC of cfDNA for the need for RRT (c, right) (AUC = 0.76 (95% CI: 0.54-0.98)).

The cfDNA levels were found to be significantly higher in those who developed severe AKI (164.8 [IQR: 139.7-200 vs. 123.0 [IQR: 100.9-137.7 ng/mL; *p* < 0.001) (Figure 1(a)) and those needing RRT (164.8 [IQR: 136.7-200 vs. 125.0 [IQR: 101.6-139.3 ng/mL; *p* = 0.020) (Figure 1(b)). The receiver operating curve (ROC) AUCs for cfDNA concentration for predicting severe AKI and need for RRT were 0.82 (95% CI 0.67-0.98, *p* < 0.001) (Figure 1(c)) and 0.76 (95% CI 0.54-0.98, *p* = 0.018) (Figure 1(c)), respectively.

### 3.3. cfDNA Levels as a Predictor for Severe AKI

In the multivariate logistic regression (adjusted for age, comorbidities, and ED SCr), each one-unit increase in cfDNA value was associated with 4.6% increased odds for development of severe AKI (OR = 1.046; 95% CI 1.002-1.092, *p* = 0.040) (Table 2).

### 3.4. Correlation between cfDNA Levels and Markers of AKI, TMA, and Inflammation

cfDNA was found to be positively correlated with ED SCr (*r* = 0.426; *p* = 0.002), NGAL (*r* = 0.545; *p* < 0.001), cystatin C (*r* = 0.330; *p* = 0.022), neutrophil count (*r* = 0.281; *p* = 0.048), neutrophil-to-lymphocyte ratio (*r* = 0.402; *p* = 0.005), C3a (*r* = 0.625; *p* < 0.001), C3a/C3 ratio (*r* = 0.620; *p* < 0.001), C5a (*r* = 0.450; *p* = 0.001), Scb5-9 (*r* = 0.462, *p* = 0.001), IL-6 (*r* = 0.665; *p* < 0.001), IL-8 (*r* = 0.462; *p* < 0.001), IL-10 (*r* = 0.462; *p* < 0.001), TNF-*α* (*r* = 0.309; *p* = 0.031), LDH (*r* = 0.563; *p* < 0.001), CRP (*r* = 0.625; *p* < 0.001), ferritin (*r* = 0.454; *p* = 0.001), and fibrinogen (*r* = 0.366; *p* = 0.020) and negatively correlated with ADAMTS13/VWF ratio (*r* = −0.433; *p* = 0.002) and lymphocyte count (*r* = −0.369; *p* = 0.011). A negative correlation was observed with platelet counts, nearly reaching statistical significance (*p* = 0.055) (Table 3 and supplemental Figure S1).

### 3.5. Correlation between cfDNA Levels and Other NETs Components

**Figure 2:**
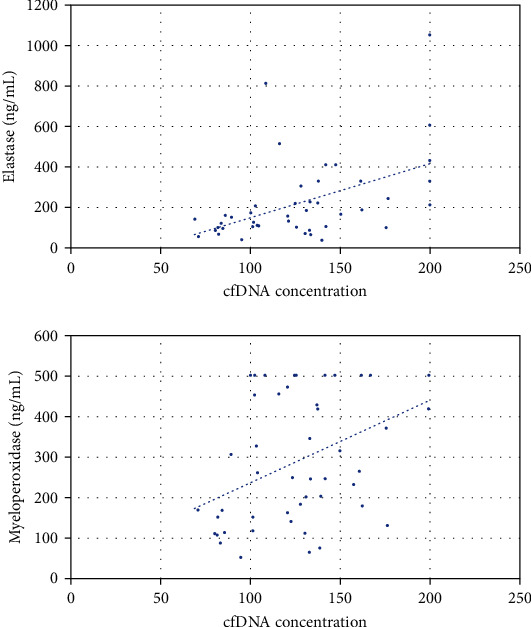
Scatter plots for cell-free DNA concentration (cfDNA) and elastase (*r* = 0.521; *p* < 0.001) and myeloperoxidase (MPO) levels (*r* = 0.438; *p* = 0.002). Dotted blue lines represent the linear association.

The cfDNA levels were found to be positively correlated with neutrophil elastase (*r* = 0.521; *p* < 0.001) and MPO (*r* = 0.438; *p* = 0.002), which are components of NETs, further validating cfDNA as a NETosis surrogate (Figure 2 and Table 3). Interestingly, neutrophil elastase was significantly higher in patients who developed severe AKI (242.6 ng/mL [IQR: 173.1-368.9]) compared to patients who did not (132.4 ng/mL [IQR: 95.8-218.4]; *p* = 0.029) (Supplemental Figures S2 and S3).

## 4. Discussion

In this prospective study, we observed an elevated cfDNA to be significantly associated with severe AKI and the need for RRT, providing further evidence that intravascular NETosis could be an important contributing factor in the development of microthrombosis and COVID-19–associated AKI. We observed that cfDNA was significantly correlated with markers of severe COVID-19 [27], including neutrophilia, elevations in proinflammatory cytokines, and over-activation of complement. Endothelial injury in COVID-19 is driven by hypercytokinemia and dysregulated immune response, which may be exacerbated by other mechanisms, such as potential direct viral infection of endothelial cells [28]. Moreover, cfDNA was correlated with other specific markers of NETosis, MPO, and neutrophil elastase. It has been suggested that NETs may serve as a pathologic bridge between endothelial injury and complement activation in other forms of TMA, such as hematopoietic cell transplant–associated TMA [29]. Most recently, NETosis was put forward as one of the pivotal culprits in the pathomechanism of end-organ damage in COVID-19 [30, 31].

We observed a negative correlation of cfDNA with the ADAMTS13:VWF ratio, a marker associated with COVID-19 severity and severe AKI, along with a positive correlation with LDH, ferritin, and haptoglobin, suggesting a picture of a secondary TMA. Multiple processes underlie the interaction between VWF, ADAMTS13, and cfDNA/NETs. In brief, NETosis would result in reduction of ADAMTS13 activity via mechanisms such as oxidation, citrullination, proteolysis, and competition for binding VWF [32]. Ultimately, this may lead to elevated VWF:Ag and propagation of ultra large VWF multimers, promoting thrombosis [32]. Such multimers may also then recruit and activate more neutrophils, enhancing NETosis and further exacerbating thromboinflammation, in a merciless loop [32]. We also observed significant positive correlations for cfDNA with complement components C3a, C5a, and Scb5-9. Given the interactions between the complement system and neutrophils, we suspect that crosstalk between these two components of innate immunity leads to complement overactivation and drives NETosis generation in a self-amplifying cycle, culminating in the activation of prothrombotic pathways and endothelial injury, thus further exacerbating thromboinflammation and tissue damage.

Ex vivo studies have demonstrated the ability of SARS-CoV-2 to activate NETosis in human neutrophils [33]. Overall, NETosis is an important immune mechanism for containment and elimination of pathogens. Viruses have been demonstrated to activate neutrophils via toll-like receptors (TLRs) 4, 7, and 8, leading to releases of reactive oxygen species and NETs, as well as disruption of viral replication via obstruction of protein kinase C pathways [34]. However, excessive NETosis can lead to microvascular thrombosis and tissue damage. Intravascular NETs can activate the contact pathway and enhance the extrinsic pathways of coagulation via TF presentation while simultaneously disrupting the functions of anticoagulants, such as antithrombin and TF pathway inhibitor [35–38].

This study was limited by a relatively small sample size, while strengthened by the rich collection of biomarkers measured in this cohort, allowing assessment of interactions of cfDNA with multiple systems. Interestingly, we found a statistically nonsignificant negative correlation between platelet count and cfDNA (*p* = 0.055). Given the borderline *p* value, we suspect that this may be related to the timing of sample measure, which was performed in this study relatively early in the disease course, at first presentation to the ED. As we suspect that platelet levels would continue to drop with disease progression, further studies evaluating these associations with samples obtained later in the course of disease, and in larger cohorts, are warranted.

Given the suspected prominent role of endothelial injury and microvascular thrombosis in the pathology and clinical outcomes of COVID-19, and that NETosis may serve as a bridge between multiple systems driving multiorgan injury, NETs may represent an appealing target for new clinical trials. To date, therapeutics for SARS-CoV-2 infection remain limited and given the multifactorial pathology of severe illness, identifying effective therapeutic targets is challenging. Indeed, drugs that interfere with NETosis, this pathologic process which may bridge multisystem injuries, such as dipyridamole [39] and heparin [40], have shown promise in early studies. Moreover, many other drugs that interfere with NETs are available [34]. Further research is urgently needed to understand the role of NETosis in COVID-19 and evaluate therapeutic avenues for targeting this process.

## 5. Conclusion

Elevated cfDNA are significantly associated with severe AKI and the need for RRT, providing further evidence that intravascular NETosis could be an important contributing factor in the development of microthrombosis and COVID-19–associated AKI. Further research is urgently needed to understand the role of NETosis in COVID-19 and evaluate therapeutic avenues for targeting this process.

## Figures and Tables

**Table 1:** Baseline demographics of the Cincinnati Emergency Department COVID-19 cohort.

Variable	All patients (*n* = 51)	KDIGO AKI stage		*p* Value
		0 + 1	2 + 3	
Age (years): median (IQR)	50.5 (41-66)	47 (37.5-64)	66 (56.5-70.2)	**0.005**
Sex (male): *n* (%)	31	24 (77.4%)	7 (22.6%)	1.000
*Race:n(%)*				
Black	20	11 (55.0%)	9 (45.0%)	**0.036**
Hispanic	19	18 (94.7%)	1 (5.3%)	
White	9	7 (77.8%)	2 (22.2%)	
Other	3	3 (100%)	0 (0%)	
*Comorbidities:n* *(%)*				
Coronary artery disease	8	3 (37.5%)	5 (62.5%)	**0.012**
Heart failure	9	3 (33.3%)	6 (66.7%)	**0.003**
Hypertension	25	14 (56.0%)	11 (44.0%)	**0.002**
Hyperlipidemia	15	11 (73.3%)	4 (26.7%)	0.730
Obesity	18	16 (88.8%)	2 (11.1%)	0.086
Diabetes	20	14 (70.0%)	6 (30.0%)	0.512
Chronic obstructive pulmonary disease	8	4 (50%)	4 (50%)	0.076
Asthma	8	6 (75%)	2 (25%)	1.000
Chronic kidney disease	6	1 (16.7%)	5 (83.3%)	**0.002**
Chronic liver disease	7	3 (42.9%)	4 (57.1%)	**0.044**
Cerebrovascular disease	1	0 (0%)	1 (100%)	0.375
Acquired immunodeficiency (HIV, transplant)	3	2 (66.7%)	1 (33.3%)	1.000
Autoimmune disease	2	2 (100%)	0 (0%)	1.000

AKI, acute kidney injury; HIV, human immunodeficiency virus; IQR, interquartile range; KDIGO, kidney disease: improving global outcomes. Bold values indicate statistical significance.

**Table 2:** Diagnostic performance of cell-free DNA (cfDNA) for predicting severe acute.

cfDNA				
	**Cutoff (ng/mL)**	**Sensitivity**	**Specificity**	**AUC (95% CI)**
Severe AKI	161.3	0.67	0.92	0.82 (0.67-0.97)
Need for RRT	142.0	0.75	0.77	0.76 (0.54-0.98)

AKI, acute kidney injury; AUC, area under the curve; CI, confidence interval; cfDNA, cell-free DNA; RRT, renal replacement therapy.

**Table 3:** Correlation between cell-free DNA (cfDNA) and markers of AKI, TMA, inflammation, and NETs components.

Correlated to cfDNA	Spearman's correlation	*p* Value
ED creatinine	0.426	0.002
NGAL	0.545	<0.001
Cystatin C	0.330	0.022
ADAMTS13	-0.209	**0.146**
VWF	0.393	0.005
ADAMTS13/VWF	-0.433	0.002
C3a	0.625	<0.001
C3a/C3	0.620	<0.001
CRP	0.625	<0.001
Ferritin	0.454	0.001
IL-6	0.665	<0.001
IL-8	0.442	0.001
IL-10	0.462	0.001
Plasminogen	0.053	**0.716**
Fibrinogen	0.366	0.020
PAI-1	0.173	**0.231**
LDH	0.563	<0.001
Angiopoietin 1	0.082	**0.576**
Angiopoietin 2	0.279	**0.052**
Lymphocytes	-0.368	0.011
Neutrophils	0.281	0.048
NLR	0.402	0.005
C5a	0.450	0.001
Sc5b9	0.462	0.001
Haptoglobin	0.508	<0.001
TNFalpha	0.309	0.031
Elastase (ng/mL)	0.521	<0.001
Myeloperoxidase (ng/mL)	0.438	0.002
Platelets	-0.282	**0.055**

AKI, acute kidney injury; cfDNA, cell-free DNA; CRP, C-reactive protein; ED, emergency department; LDH, lactate dehydrogenase; NGAL, neutrophil gelatinase–associated lipocalin; VWF, von-Willebrand factor.

## Data Availability

The datasets generated during and/or analyzed during the current study are not publicly available due to patient privacy and confidentiality, but are available from the corresponding author on reasonable request.

## Supplementary Materials

Supplementary Materials Supplemental Figure S1: scatter plots for cell-free DNA (cfDNA) concentration and TMA biomarkers. Dotted blue lines represent linear association. cfDNA was found to be positively correlated with C3a (*r* = 0.625; *p* < 0.001), C3a/C3 ratio (*r* = 0.620; *p* < 0.001), Scb5-9 (*r* = 0.462, *p* = 0.001), TMAIL-6 (*r* = 0.665; *p* < 0.001), LDH (*r* = 0.563; *p* < 0.001), ferritin (*r* = 0.454; *p* = 0.001), fibrinogen (*r* = 0.366; *p* = 0.020), VWF (*r* = 0.393; *p* = 0.005), and haptoglobin (HAPT) (*r* = 0.508; *p* < 0.001?) and negatively correlated with ADAMTS13 (*r* = −0.433; *p* = 0.002). Supplemental Figure S2: association between levels of and Elastase and MPO measured upon presentation to the ED with development of severe AKI or need for RRT. While only elastase levels were significantly different according to development of severe AKI (A), levels of both MPO and elastase showed a nonstatistically significant trend toward both development of severe AKI and need for RRT (B, C, D). Supplemental Figure S3: receiver operating characteristic curve (ROC) of elastase and myeloperoxidase (MPO) for the development of severe AKI or need for RRT. For Elastase, area under the curve (AUC) =0.72 (95% CI: 0.55-0.89) for need for severe AKI and (AUC) = 0.7 (95% CI: 0.53-0.87) for need for RRT (A, B); For MPO, area under the curve (AUC) = 0.602 (95% CI: 0.4-0.82) for need for severe AKI and AUC = 0.71 (95% CI: 0.49-0.93) for need for RRT (C, D).
