# Supplementary material for: Cell-Free DNA, Neutrophil extracellular traps (NETs), and Endothelial Injury in Coronavirus Disease 2019– (COVID-19–) Associated Acute Kidney Injury
Source: Mediators Inflamm. 2022 Apr 22;2022:9339411. doi: 10.1155/2022/9339411 (PMC9054425; doi:10.1155/2022/9339411)
Supplement: Supplementary Materials — Supplemental Figure S1: scatter plots for cell-free DNA (cfDNA) concentration and TMA biomarkers. Dotted blue lines represent linear association. cfDNA was found to be positively correlated with C3a (r = 0.625; p < 0.001), C3a/C3 ratio (r = 0.620; p < 0.001), Scb5-9 (r = 0.462, p = 0.001), TMAIL-6 (r = 0.665; p < 0.001), LDH (r = 0.563; p < 0.001), ferritin (r = 0.454; p = 0.001), fibrinogen (r = 0.366; p = 0.020), VWF (r = 0.393; p = 0.005), and haptoglobin (HAPT) (r = 0.508; p < 0.001?) and negatively correlated with ADAMTS13 (r = −0.433; p = 0.002). Supplemental Figure S2: association between levels of and Elastase and MPO measured upon presentation to the ED with development of severe AKI or need for RRT. While only elastase levels were significantly different according to development of severe AKI (A), levels of both MPO and elastase showed a nonstatistically significant trend toward both development of severe AKI and need for RRT (B, C, D). Supplemental Figure S3: receiver operating characteristic curve (ROC) of elastase and myeloperoxidase (MPO) for the development of severe AKI or need for RRT. For Elastase, area under the curve (AUC) =0.72 (95% CI: 0.55-0.89) for need for severe AKI and (AUC) = 0.7 (95% CI: 0.53-0.87) for need for RRT (A, B); For MPO, area under the curve (AUC) = 0.602 (95% CI: 0.4-0.82) for need for severe AKI and AUC = 0.71 (95% CI: 0.49-0.93) for need for RRT (C, D). [file 9339411.f1.docx]

***Research Article***

***Cell-free DNA, neutrophil extracellular traps (NETs), and endothelial injury in Coronavirus disease 2019 (COVID-19)-associated Acute Kidney Injury***

Brandon Michael Henry^a,b^, Maria Helena Santos de Oliveira^c^, Isaac Cheruiyot^d^, Justin Benoit^e^, James Rose^b^, Emmanuel J. Favaloro^f^, Giuseppe Lippi^g^, Stefanie Benoit^b,h,i^, Naomi Pode Shakked^b,j,k^

^a^ The Heart Institute, Cincinnati Children’s Hospital Medical Center, Cincinnati, (OH,) USA

^b^ Division of Nephrology and Hypertension, Cincinnati Children’s Hospital Medical Center, Cincinnati, (OH,) USA

^c^ Department of Statistics, Federal University of Parana, Curitiba, Brazil

^d^ School of Medicine, University of Nairobi, Kenya

^e^ Department of Emergency Medicine, University of Cincinnati, Cincinnati, (OH,) USA

^f^ Haematology, Sydney Centres for Thrombosis and Haemostasis, Institute of Clinical Pathology and Medical Research (ICPMR), NSW Health Pathology, Westmead Hospital, Westmead, NSW, Australia

^g^ Section of Clinical Biochemistry, Department of Neurosciences, Biomedicine and Movement Sciences, University of Verona, Italy

^h^ Division of Bone Marrow Transplantation and Immunodeficiency, Cincinnati Children’s Hospital Medical Center, Cincinnati, (OH,) USA

^i^ Department of Pediatrics, University of Cincinnati College of Medicine, Cincinnati, Ohio, USA

^j^ The Sheba Talpiot Medical Leadership Program, Sheba Medical Center, Israel

^k^ Sackler School of Medicine, Tel Aviv University, Tel Aviv, Israel

Short Title: NETs in COVID-19 associated AKI

Corresponding Author:

Naomi Pode Shakked, MD PhD

Division of Nephrology and Hypertension

Cincinnati Children’s Hospital Medical Center

3333 Burnet Ave.

Cincinnati, OH, USA 45229

E-mail: [naomi.podeshakked@cchmc.org](mailto:naomi.podeshakked@cchmc.org)

**Supplemental data:**

Supplemental Figures 2

Figure S1 3

Figure S2 4

Figure S3 5

#### Figure S1

####
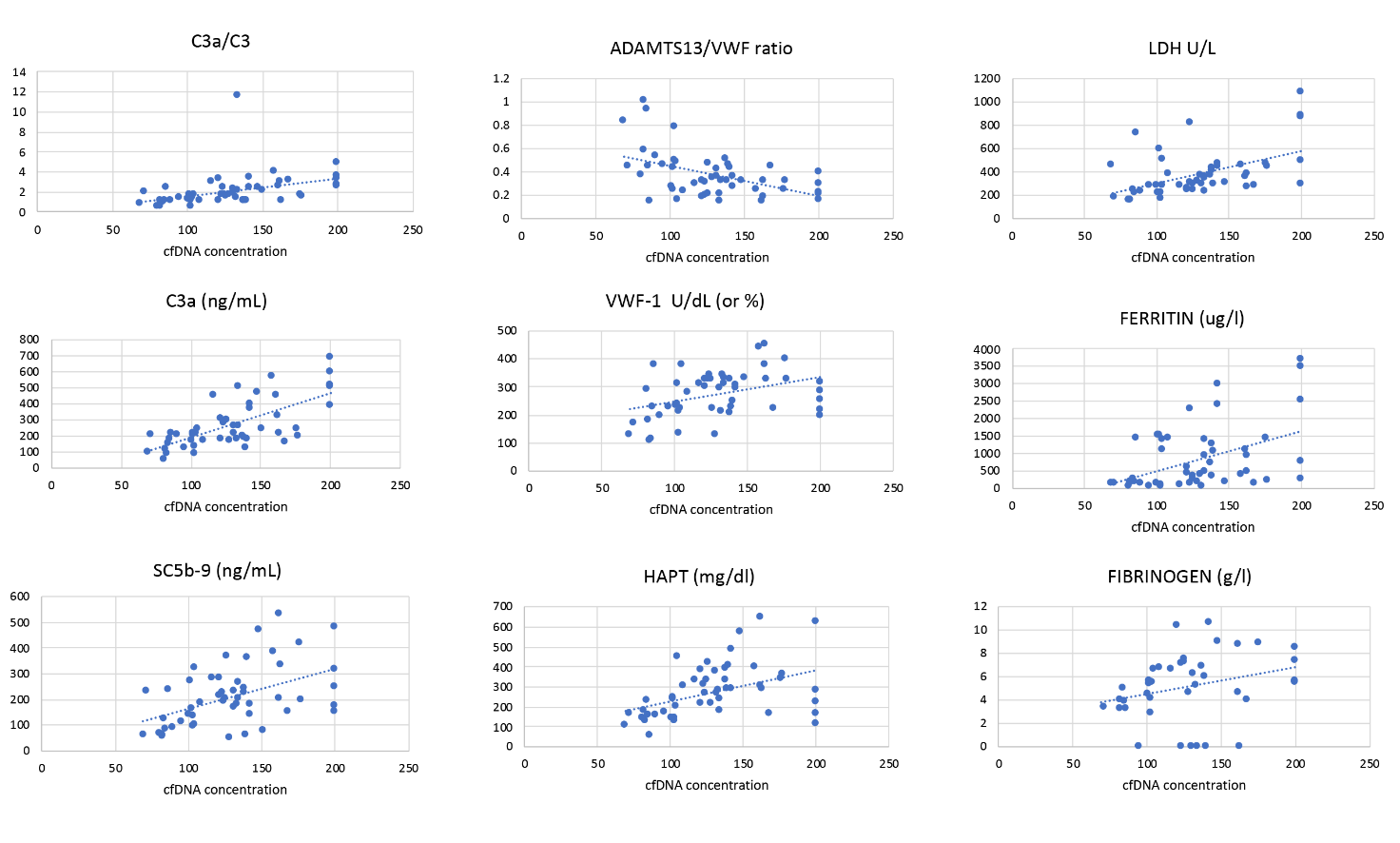


#### Scatter plots for Cell free DNA (cfDNA) concentration and TMA biomarkers. Dotted blue lines represent linear association. cfDNA was found to be positively correlated with C3a (r=0.625; p<0.001), C3a/C3 ratio (r=0.620; p<0.001), Scb5-9 (r=0.462, p=0.001), TMAIL-6 (r=0.665; p<0.001), LDH (r=0.563; p<0.001), ferritin (r=0.454; p=0.001), fibrinogen (r=0.366; p=0.020), VWF (r=0.393; p=0.005), Haptoglobin (HAPT) (r=0.508; p<0.001) and negatively correlated with ADAMTS13 (r=-0.433; p=0.002).

####
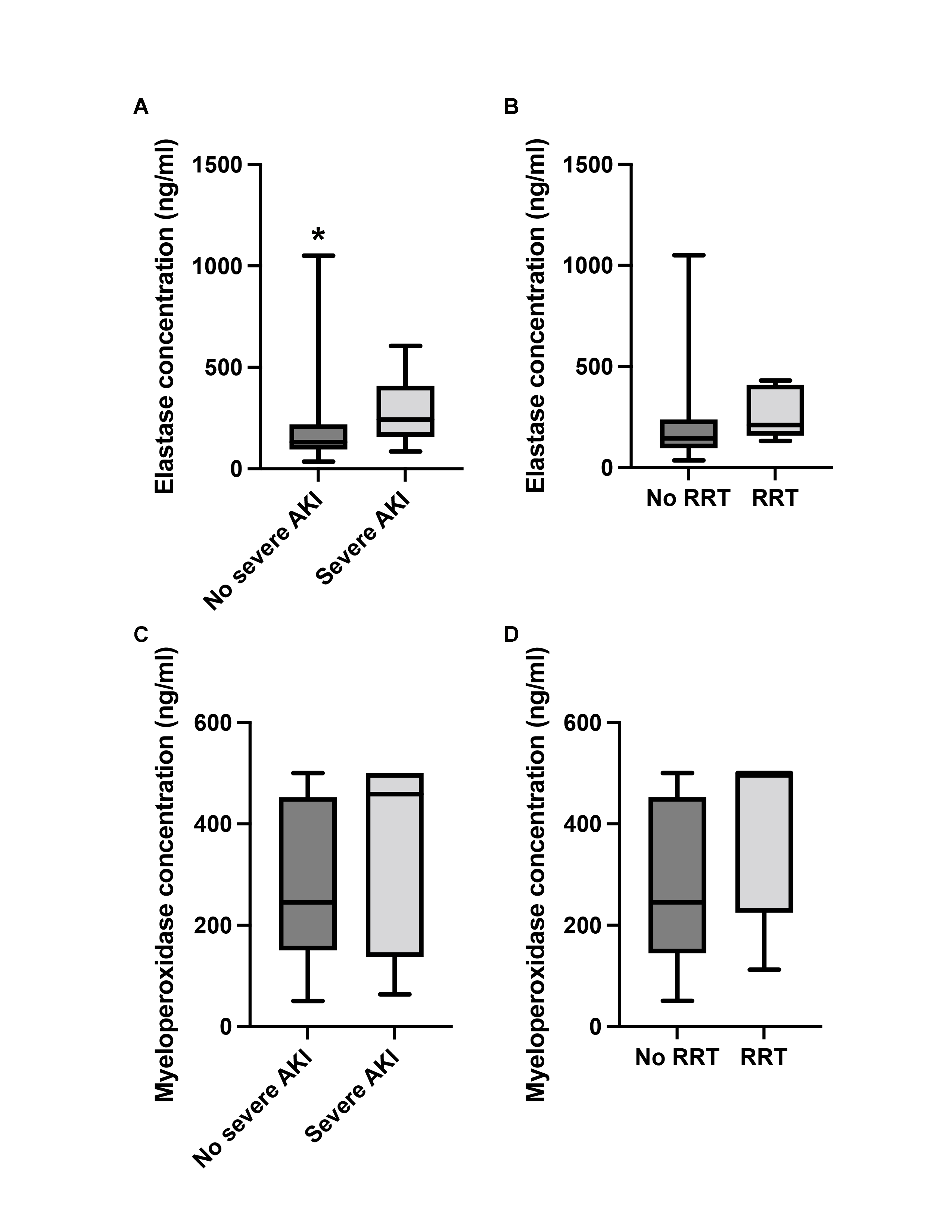
Figure S2

Association between levels of and Elastase and MPO measured upon presentation to the ED with development of severe AKI or need for RRT. While only Elastase levels were significantly correlated different according to with development of severe AKI (A), levels of both MPO and Elastase showed a non-statistically significant trend toward both development of severe AKI and need for RRT (B, C, D).

####
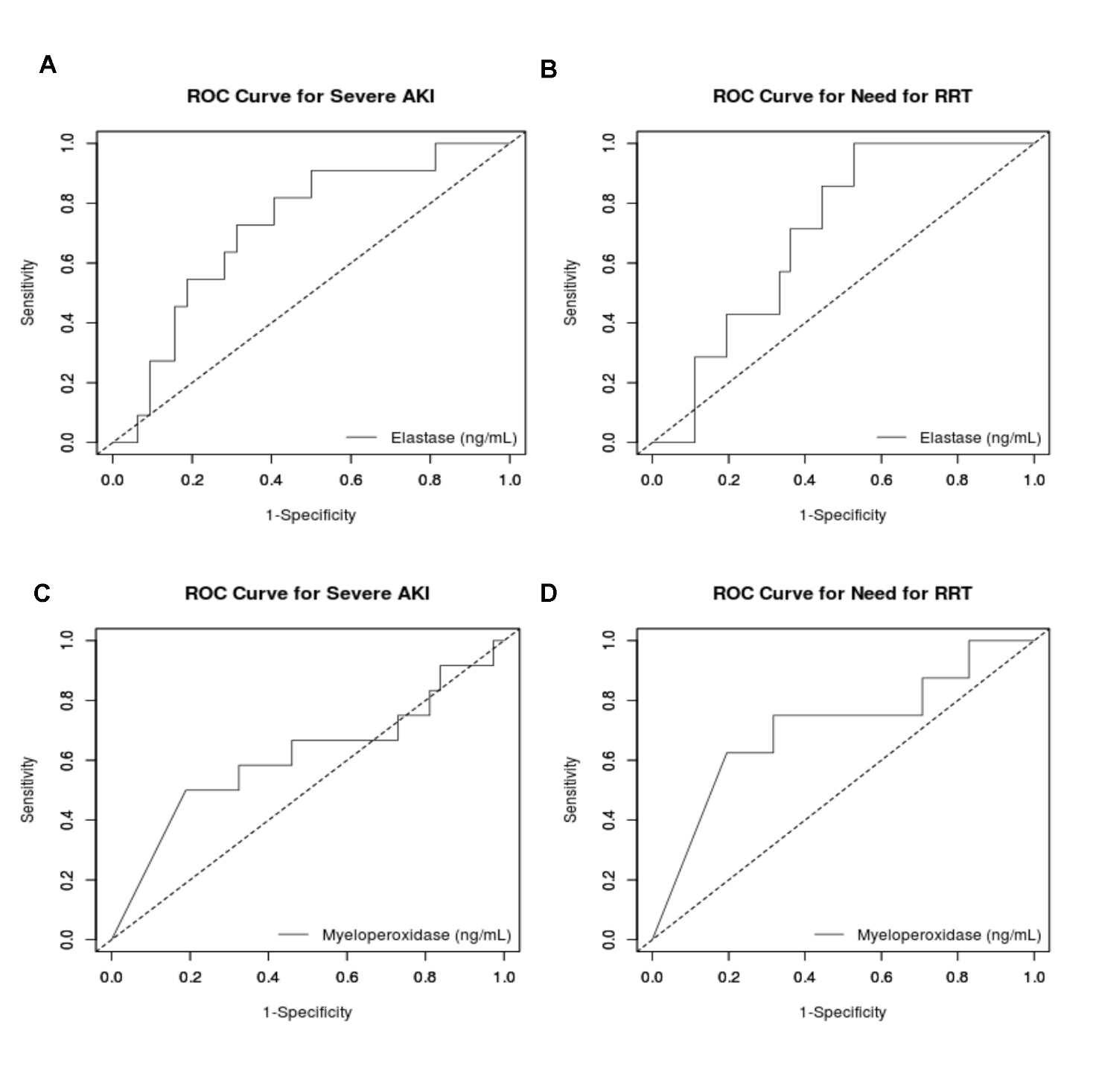
Figure S3

#### Receiver operating characteristic curve (ROC) of Elastase and Myeloperoxidase (MPO) for development of severe AKI or need for RRT. For Elastase, area under the curve (AUC)=0.72 (95%CI: 0.55-0.89 for need for severe AKI and (AUC)=0.7 (95%CI: 0.53-0.87)) for need for RRT (A, B); For MPO, area under the curve (AUC)=0.602 (95%CI: 0.4-0.82 for need for severe AKI and AUC=0.71 (95%CI: 0.49-0.93)) for need for RRT (C, D).
